# Endo-aortic balloon occlusion for reoperative minimally invasive mitral valve replacement on intra-aortic balloon pump: a case report

**DOI:** 10.1051/ject/2025023

**Published:** 2025-12-17

**Authors:** Jenna L. Cornibe, Vivek Patel, Austin Erney

**Affiliations:** 1 Jupiter Medical Center 1210 S Old Dixie Hwy Jupiter FL 33458 USA; 2 Perfusion Services, Epic Specialty Staffing 2250 McGregor Blvd Suite 3300 Fort Myers FL 33901 USA

**Keywords:** Minimally invasive, Endoballoon, Endo-aortic balloon occlusion, EABO, IABP

## Abstract

Minimally invasive approaches are the future of cardiac surgery. Coupled with that are devices such as endo-aortic balloon occlusion (EABO) which allow the success of such procedures. EABO has also proven invaluable in high-risk or reoperative cases. We report a severely high-risk patient who presented for reoperative minimally invasive mitral valve replacement with an intra-aortic balloon pump (IABP) and multiple high-dose vasopressors. The EABO was utilized over a transthoracic cross clamp (TCC). With careful maneuvering upon ascent into the aorta, the deflated endoballoon easily passed beyond the deflated IABP balloon under transesophageal echocardiogram (TEE) guidance. After completion of arrest, surgical placement of the bioprosthetic valve, and spontaneous return of cardiac rhythm once EABO was completed, the deflated endoballoon was carefully retracted back down through the aorta past the deflated IABP balloon. The integrity of the IABP balloon remained intact and was able to resume function once cardiopulmonary bypass (CPB) was terminated. We found that EABO was a safe and effective alternative technique for aortic occlusion in our patient supported on IABP with previous sternotomy.

## Introduction

It is widely understood that modern medicine has shown innumerable advances throughout the years. Not only have surgical techniques become minimally invasive, but coupled with that come the tools and products that have allowed this progression. Cardiac surgery is not immune to this evolution. Full sternotomy with transthoracic cross-clamp (TTC) is still the most common approach to open-heart procedures. However, recent advancements in minimally invasive and robotic-assisted cardiac surgery in combination with the Edwards ThruPort^TM^ Systems IntraClude^TM^ (Edwards Lifesciences LLC, Irvine, CA, USA) provide alternative options to these. Minimally invasive and robotic-assisted cardiac surgeries continue to surge in utility as they are not only aesthetically pleasing to the patient but can also offer less post-operative discomfort and length of recovery. However, smaller incisions yield the surgeon less visual and working area. The IntraClude^TM^, an endo-aortic balloon occlusion (EABO) device, provides an internal approach for aortic occlusion, cardioplegia delivery, and venting for such cases.

Severe mitral valve regurgitation (MR) can present with congestive heart failure and dysfunction of the left ventricle (LV) [1]. A study by Dekkar et al. showed that the use of an intra-aortic balloon pump (IABP) decreases MR and increases output directed toward the aorta without functional change of the LV, due to a decrease in aortic impedance [2].

We report a complex reoperative severe MR patient on IABP in which the EABO provided an invaluable alternative for arrest and cardioplegia delivery.

## Case report

A 63-year-old, 170 cm, and 70 kg male patient presents with chronic kidney disease, hyperlipidemia, and an ejection fraction of 40%. Additionally, he has an old myocardial infarction, ischemic heart failure, and ventricular tachycardia. Past interventions include five-vessel coronary artery bypass grafting, which occurred 29 years prior, as well as having an implantable cardioverter defibrillator 3 years prior. He had no known drug allergies. The day before surgery, the patient had an attempted MitraClip^TM^ (Abbott, Santa Clara, CA, USA) x2 for severe MR, in which he had been symptomatic for the past year. This procedure failed, resulting in worsening leaflet prolapse with new ruptured chordae, worsened leaflet flail, and worse MR. This outcome rendered the patient hemodynamically unstable, requiring increasing medical support with three vasopressors and the addition of an IABP (inserted via the left common femoral artery). The patient’s Society of Thoracic Surgeons (STS) risk score for operative mortality was 19.7%.

The critically ill patient was brought to the operating room for minimally invasive mitral valve replacement (previous sternotomy), supported by IABP and vasopressors. Right and left radial arterial lines were placed. Oximetry sensors were utilized cerebrally as well as on the lower calves. Once prepped and draped, incisions were made in the right common femoral artery and vein for cannulation. The prepped IntraClude^TM^ system pressure lines were attached to an anesthesia pressure module for the aortic root pressure reading, and a pressure line to the cardioplegia system pressure module for the intra-aortic endoballoon pressure reading. Both lines were flushed and de-aired per protocol. Right anterior thoracotomy was performed, and lung adhesions were divided. The patient did not tolerate single lung ventilation, and therefore, we proceeded with cannulation.

The patient was systemically anticoagulated with 22,000 Units (U) heparin, and a 23 French (Fr) Edwards ThruPort^TM^ Systems EndoReturn^TM^ Arterial Cannula (Edwards Lifesciences LLC, Irvine, CA, USA) was placed in the right common femoral artery along with a distal femoral artery perfuser. A 25 Fr Medtronic Bio-Medicus^TM^ NextGen cannula (Medtronic, Minneapolis, MN, USA) was introduced through the right femoral vein up to the superior vena cava. Through the bifurcation of the EndoReturn^TM^ cannula, the endoballoon was introduced. Once the activated clotting time (ACT) reached 480 seconds (s), cardiopulmonary bypass (CPB) was initiated while simultaneously stopping/deflating the IABP. With transesophageal echocardiogram (TEE) guidance, the deflated endoballoon was carefully advanced past the deflated IABP balloon and into the appropriate position within the ascending aorta. Aortic root venting was started and the endoballoon was then inflated per protocol. The initial endoballoon pressure reading was 480 mmHg. Venting stopped and 2000 mL of 4:1 Del Nido antegrade cardioplegia at 4 °C was given for the arrest. The patient’s core temperature drifted to 35 °C during the EABO time frame. The native valve was excised along with both MitraClips^TM^ and replaced with a 33 mm bioprosthetic valve. De-airing procedures and aortic root venting commenced, and the endoballoon was deflated. The average endoballon pressure throughout inflation was 395 mmHg. Although the patient regained spontaneous cardiac rhythm, he was pacer-dependent at a low threshold of 40 bpm, therefore, the pacer representative increased the rate to 80 bpm. During this time of attempted weaning, the patient’s oxygenation was poor and required bronchoscopy to clear excess pulmonary secretions related to pulmonary edema. He also received 2 units of packed red blood cells and was started on nitric oxide. The endoballoon was retracted to the EndoReturn^TM^ cannula. CPB was terminated, and simultaneously the IABP was restarted at a timing ratio of 1:1. CPB time was 205 min, and EABO time was 67 min. A total volume of 1500 mL of hemoconcentration was removed throughout the CPB run. Heparinization was reversed with protamine sulfate. Both sets of oximetry sensors remained unchanged during the entire perioperative period. The patient returned to the intensive care unit on IABP, high-dose inotropic support, and nitric oxide.

## Discussion

Reoperative cardiac patients bring a different subset of challenges when deciding upon an approach for surgery. This patient not only had a previous sternotomy but he was also supported on IABP and multiple high-dose vasopressors, which deemed him severely high-risk. Preoperative discussion regarding arrest techniques favored EABO over fibrillating heart due to associated risks, one of which is an increasingly high for stroke within the perioperative time frame [3]. The usage of an EABO device can be paramount for such reoperative sternotomy procedures. Barbero et al. published a study comparing the usage between EABO and TTC, showing a decrease in bleeding during the post-operative time frame with the EABO group, even though a multitude were higher acuity reoperative cases [4]. This study also equated both the efficacy and safety of TCC and EABO [4]. A right anterior thoracotomy minimally invasive approach was also decided to provide a safer opening versus a re-do sternotomy. Communication during the progression of the procedure was vital as cannulation, IABP, and the IntraClude^TM^ system were all introduced through femoral vessels and had the potential to counteract. The IntraClude^TM^ system and its components were standardly prepped. Careful consideration had to be taken when introducing the deflated endoballoon past the deflated IABP balloon, particularly to avoid a puncture, as it was reasonably assumed with this patient’s presentation that it would be required post-CPB. For this reason, the IABP balloon was left in place throughout the CPB run instead of retracting out of the aorta. The TEE guidance assisted with this, as with all our IntraClude^TM^ utilized cases. Once the deflated endoballoon was extended safely beyond the deflated IABP balloon, the case proceeded otherwise as normal. The patient’s left internal mammary artery (LIMA) graft to the left anterior descending (LAD) artery was no longer patent from the previous surgery, and therefore, there was no issue with inducing or sustaining cardiac arrest. After the surgical valve was in place the position and function were verified under TEE and the arrest period was complete, de-airing/venting occurred before deflating the endoballoon. Careful consideration was again taken to retract the deflated endoballoon down past the deflated IABP balloon in the descending aorta, retracting it to the port of the EndoReturn^TM^ cannula. At this point, we were able to terminate CPB and safely resume IABP support. EABO was a safe and efficient alternative technique for aortic occlusion in our patient with a previous sternotomy supported by IABP. As stated in Rosen and Guy, the future mitral valve surgeon and EABO go hand-in-hand [5].

## Data Availability

All available data are incorporated into the article.
